# EC: an efficient error correction algorithm for short reads

**DOI:** 10.1186/1471-2105-16-S17-S2

**Published:** 2015-12-07

**Authors:** Subrata Saha, Sanguthevar Rajasekaran

**Affiliations:** 1 Department of Computer Science & Engineering, University of Connecticut, 371 Fairfield Way, Unit 4155, Storrs, CT 06269-4155, USA

## Abstract

**Background:**

In highly parallel next-generation sequencing (NGS) techniques millions to billions of short reads are produced from a genomic sequence in a single run. Due to the limitation of the NGS technologies, there could be errors in the reads. The error rate of the reads can be reduced with trimming and by correcting the erroneous bases of the reads. It helps to achieve high quality data and the computational complexity of many biological applications will be greatly reduced if the reads are first corrected. We have developed a novel error correction algorithm called EC and compared it with four other state-of-the-art algorithms using both real and simulated sequencing reads.

**Results:**

We have done extensive and rigorous experiments that reveal that EC is indeed an effective, scalable, and efficient error correction tool. Real reads that we have employed in our performance evaluation are Illumina-generated short reads of various lengths. Six experimental datasets we have utilized are taken from sequence and read archive (SRA) at NCBI. The simulated reads are obtained by picking substrings from random positions of reference genomes. To introduce errors, some of the bases of the simulated reads are changed to other bases with some probabilities.

**Conclusions:**

Error correction is a vital problem in biology especially for NGS data. In this paper we present a novel algorithm, called *Error Corrector (EC)*, for correcting substitution errors in biological sequencing reads. We plan to investigate the possibility of employing the techniques introduced in this research paper to handle insertion and deletion errors also.

**Software availability:**

The implementation is freely available for non-commercial purposes. It can be downloaded from: http://engr.uconn.edu/~rajasek/EC.zip.

## Introduction

In sequencing technology numerous small fragments are generated by shredding DNA molecule in random positions. Followed by this, the chain termination method is used to obtain reads from these small-sized fragments. The above procedures are applied repeatedly to get multiple overlapping reads. By exploiting overlap information the resulting reads are then reassembled into their original order to obtain the whole genomic sequence. This final step is done by an assembler based on the overlap graph generation and ultimately it produces a complete and continuous sequence. But the coverage of the reads in some specific regions of the genome can be low and again the reads can be erroneous due to the limitation of the NGS technologies. These events in turn produce a gap and hence the resulting overlap graph will be clustered into multiple disconnected components. As a consequence, sequence assemblers typically produce multiple unordered subsequences (i.e., contigs) instead of a complete and continuous sequence. Obtaining the exact orientation and precise order of the contigs is a very challenging and computationally intensive task. This step is known as scaffolding. If the errors can be removed from the sequencing reads, the number of contigs produced by an assembler will also be very small. In turn, the scaffolding step will be more accurate and less time consuming.

The input to the error correction problem is a large set of reads. The output should be the same set of reads with errors corrected. Depending on the NGS technology used, the length of the reads can either be fixed or variable within a certain range. There may be three types of errors that can be introduced by the sequencer. These are substitution, insertion, and deletion. One of the most popular sequencers currently is Illumina/Solexa. This sequencer has only substitution errors. In our algorithm we consider only substitution errors in the reads. In fact, most of the algorithms proposed in the literature assume only substitution errors. Coverage of sequencing is defined as the number of times a particular position of a DNA molecule is sequenced. Let the size of the genomic sequence of interest be *q *and the total length of all the reads be *q*'. The coverage can be thought of as $\frac{q\prime }{q}$. In any error correction method, coverage plays a crucial role. A high coverage normally leads to a better accuracy in error correction. In summary, we can formulate the problem of error correction in a generic way as follows: we are given a set of adequately overlapping reads. We do not know which of the reads are erroneous. The error correction problem is to identify and correct the reads that contain errors.

In this article we propose an effective, efficient, and scalable error correction algorithm called EC (Error Corrector) to correct the errors introduced by NGS technologies. To correct any read *R*, we need reads that come from the same region of the genome as *R*. Specifically, if we have many reads that sufficiently overlap with *R*, we could align each such read with *R*. Each position in *R *can then be corrected using the consensus for each position. Indeed, this is the basic theme used in any error correction algorithm. We also utilize this basic theme. Error correction algorithms found in the literature differ in how this theme is interpreted and implemented. To begin with EC builds *k*-mers from the given set of reads. Each of the *k*-mers is then hashed to a unique bucket. If two reads overlap significantly, then they are expected to share at least one *k*-mer. Thus we expect that the reads that fall into the same bucket will have a large enough overlap and possibly come from the same region of the genome. Reads from the same bucket are then aligned using our **greedy alignment **algorithm and corrected using consensus.

## Background

Correction of short biological sequencing reads is a very critical task. Many algorithmic techniques to correct short reads generated from NGS platforms can be found in the current literature. Based on the techniques used in correcting errors we can classify them into three types: *k*-spectrum based, suffix tree/array based, and multiple sequence alignment (MSA)-based. In *k*-spectrum based techniques the reads are at first decomposed into overlapping substrings of length *k*. Each substring is called a *k*-mer and the set of all *k*-mers is termed as *k*-spectrum [1]. The first *k*-spectrum based error correction algorithm has been built into the assembly tool Euler SR [1,2]. It uses a spectral alignment method where it deduces a spectrum of trusted (i.e., most probably true) *k*-mers from the input data and then corrects each read in such a way that every read contains only sequences from the spectrum. According to [1] a *k*-mer is considered solid if its multiplicity exceeds a predefined threshold and insolid otherwise. Reads containing insolid *k*-mers are then transformed to solid *k*-mers using a minimum number of edit operations. [3] follows a variant of this mechanism to correct erroneous reads. [4] presents a parallel algorithm to correct erroneous reads. This algorithm is based on spectral alignment proposed by [1] and [2] and uses the CUDA programming model. Quake [5] applies the same *k*-mer spectrum framework as described above. In addition, it introduces quality values and rates of specific miscalls computed from each sequencing project. It calculates an appropriate coverage cutoff between trusted and erroneous *k*-mers. It is based on calculating the weight of a *k*-mer as the weighted sum of all its instances, i.e., bases using the quality values assigned to each base.

Reptile [6] also incorporates the *k*-mer spectrum approach and exploits quality information of bases when available. It corrects errors by simultaneously examining possibilities to correct erroneous reads employing a Hamming distance-based approach and contextual information between neighboring reads. The algorithmic approach of [7] is very similar to Reptile. At first it sorts the *k*-mers to find the set of distinct *k*-mers and also the multiplicity of each distinct *k*-mer. It then constructs the Hamming graph and then finds the connected components from the graph. Each connected component is termed as a cluster. Each cluster is then processed to find a consensus string and erroneous reads are corrected based on the consensus string. Musket [8] is an efficient multi-stage *k*-mer based corrector for Illumina short-read data. It employs Bloom filter to count the number of occurrences of all non-unique *k*-mers. To correct errors Musket employs three mechanisms namely two-sided conservative correction, one-sided aggressive correction, and voting-based refinement. Another *k*-spectrum based error correction tool is RACER [9]. In RACER a predefined threshold *t *is introduced to correct errors. A nucleotide *a* following a *k*-mer is assumed correct if the count of the *k*-mer is ≥ *t *and erroneous otherwise.

In the alignment approach, multiple alignments are computed for the probable aligned reads. The errors are then detected and corrected based on the columns of the alignment. Some of the early error correction tools using multiple alignments include MisEd [10] and Arachne [11]. Coral [12] and ECHO [13] are two of the most recently developed multiple alignments based techniques. Coral starts by indexing all the *k*-mers and their reverse complements. It records each *k*-mer and a list of reads associated with the *k*-mer by creating a hash table. After indexing, each read is taken as a base read and is aligned with other reads that share at least one *k*-mer with the base read. Needleman-Wunsch algorithm is used for alignment. A problem with any alignment-based approach is that it is a computationally very expensive procedure. SHREC [14] and HiTEC [15] avoid the computation of multiple alignments by traversing a suffix tree/array data structure. SHREC is based on the generalized suffix tree. On the contrary, HiTEC is based on a more space efficient suffix array data structure. One of the variants of SHREC is Hybrid-SHREC [16].

## Materials and methods

Any read *R *can be corrected using reads that sufficiently overlap with *R*. These overlapping reads can be aligned to *R *and each character in *R *can then be corrected using consensus. If the number of overlapping reads is large and the error rate is low, one would expect that the number of incorrect characters in any column in the alignment will be very small and hence the consensus will indeed be the correct character. To identify overlapping reads, we employ hashing based on *k*-mers (for a suitable value of *k*). Next we describe our error correcting algorithm EC. There are 3 main basic steps of EC:

### Candidate neighbors generation

At the beginning, for each read we identify a set of reads coming from the same genomic region with a high confidence. This is done by employing a hashing scheme. Specifically, we generate *k*-mers in each of the reads and hash the reads using the *k*-mers. As a consequence, we can expect that similar reads fall into the same hash bucket. In this approach any read *R *will be hashed into at most *r *- *k *+ 1 entries (i.e., buckets) of the hash table where *r *= |*R*|, the length of the read. For every read *R *we collect reads from the buckets that *R *falls into. Reads from the same buckets where at least one *k*-mer of *R *falls will be candidate neighbors of *R*.

### True neighbors selection

Since candidate neighbors of each read *R *are identified by considering at least one identical *k*-mer shared between *R *and its candidate neighbors, some of those neighbors may not come from the same genomic region. In this step we discard those candidate neighbors that are not likely to be true neighbors of *R*. True neighbors are those candidate neighbors of *R *that come from the same genomic region with high probability. This is the most time consuming step in the EC algorithm. Let *R *be any read and let *R*' be the candidate neighbor that has a sufficient overlap with *R*. In this elimination step we compute the Hamming distance between the two reads in the overlapping region. If this distance is greater than a threshold, we eliminate *R*' from the neighbor list of *R*. If not, we keep *R*' as a true neighbor of *R*. It is to be noted that the two reads from the same genomic region might fall into more than one buckets together. In this case we identify and use the largest overlap between the pair.

### Alignment and correction

If *R *is any read and *T *(*R*) is the list of true neighbors of *R*, we correct *R *using *T *(*R*). We align every *R*' (from T (*R*)) with *R *using our greedy alignment algorithm [17]. After aligning the reads in *T *(*R*) with *R*, we correct *R *using consensus. Specifically, let *i *be any position of *R*. We observe the characters in the reads of *T *(*R*) that occur in the same position and from out of these characters we pick the consensus (i.e., the character that occurs the most). The consensus character is used to correct the character in position *i *of *R*. Note that in this step both corrected and uncorrected reads are used to perform the correction. A corrected read is called *perfect *if the correction procedure could not find any incorrect bases. Specifically, let *R *be any read. Then *T *(*R*) will have both corrected and uncorrected reads. We align all the reads of *T *(*R*) with *R*. Since we can be more confident that the perfect reads are error free, we give a larger weight for perfect reads than other reads while correcting *R*. For example, while correcting a specific position of *R*, we look at all the characters in the reads of *T *(*R*) that occur in the same position. From out of these characters, any character from a read which is not perfect will be given a weight of 1 and any character from a perfect read will be given a weight of *w *(for some *w *> 1), while calculating the consensus character for this position. When the coverage is high we choose a smaller value for *w* than when the coverage is low. Steps of the algorithm are shown in Algorithm 1.

**Algorithm 1:** EC

**Input:** A set *S *of reads

**Output:** A set *S*' of corrected reads

1     Generate *k*-mers of each read and hash the reads based on these *k*-mers. Equal *k*-mers fall into the same bucket. If *R *is any read, any other read that falls into at least one of the buckets that *R *falls into is treated as a candidate neighbor of *R*. For every read *R *create a list *C*(*R*) of candidate neighbors.

2     Let *R *be any read. Align every read in C(*R*) with *R*. Let *R*' be any read in *C*(*R*). If *R *and *R*' overlap sufficiently and if in the overlapping region the Hamming distance between *R *and *R*' is small, then we treat *R*' as a true neighbor of *R*. For every read *R *construct a list T (*R*) of neighbors of *R *in this fashion.

3     Let *R *be any read. *R *is corrected using the reads in *T *(*R*). Greedily align *R*' with *R *for every *R*' ∈ *T*(*R*). *R *is corrected by taking a consensus across every column in the alignment. Perform this step for every read *R*.

### Complexity analysis

In this section we analyze the time complexity of EC. Let *n *be the number of reads, *r *be the read length, and *c* be the coverage. In the first step of EC, we build hash tables and generate the candidate neighbors. The number of *k*-mers generated from each read is *r *- *k *+ 1. Let *h*(.) be the has function employed. We think of the hash table as an array of buckets (or lists). Each bucket has an integer as its index. If the size of the array is *N*, then the index of any bucket is an integer in the range [1, *N*]. The expected size of each bucket is $\frac{\left(r-k+1\right)n}{N}=O\left(\frac{rn}{N}\right)$. The total time spent in building the hash table is *O*(*rn*). After constructing the hash table we find candidate neighbors of each read. A read falls into at most *r *- *k *+ 1 <*r *buckets and hence the expected number of candidate neighbors for each read is $O\left(\frac{r^{2}n}{N}\right)$. For every bucket we spend an expected $O\left({\left(\frac{rn}{N}\right)}^{2}\right)$ time. Thus the total time spent in finding candidate neighbors has an expected value of $O\left(\frac{r^{2}n^{2}}{N}\right)$.

In Steps 2 and 3 we find true neighbors and align reads. Specifically, if *R *is any read and *C*(*R*) is the list of candidate neighbors of *R*, then the expected size of *C*(*R*) is $O\left(\frac{r^{2}n}{N}\right)$. For every read *R*' ∈ *C*(*R*), we align *R*' with *R *and compute the Hamming distance between *R *and *R*' in the overlapping region. Thus for every *R*' ∈ *C*(*R*) we spend *O*(*r*) time. As a result, the total time spent in Step 3 for each read is expected to be $O\left(\frac{r^{3}n}{N}\right)$. Summing this over all the reads, the total expected time spent in Step 2 and 3 is $O\left(\frac{r^{3}n^{2}}{N}\right)$.

In summary, the expected run time of EC is $O\left(rn+\frac{r^{3}n^{2}}{N}\right)$.

### Probabilistic analysis

In this section we provide a probabilistic analysis for the effectiveness of EC. Consider a random model for the genomic sequence. Such models have been employed by others in their analyses as well. See e.g., [15]. In particular, assume that each character of the genome *G *has been uniformly and randomly picked from {*g, c, t, a*}. When we hash a read *R *based on its *k*-mers, all the neighbors of *R *should also fall into the same buckets. Also, the number of reads that are not neighbors of *R *and that fall into the same bucket as *R *should be small.

Let *x *be a *k*-mer of *R*. Let *R*' be any read that is not a neighbor of *R*. Let *y *be any *k*-mer in *R*'. We can compute the probability that *R *and *R*' fall into the same bucket as follows. If *x *and *y *are two random *k*-mers, probability that they are equal is ${\left(\frac{1}{4}\right)}^{k}$. The probability that *x *is the same as one of the *k*-mers of *R*' is thus ≤ $r{\left(\frac{1}{4}\right)}^{k}$. Also, the probability that *R *and R' will share at least one *k*-mer is ≤ $r^{2}{\left(\frac{1}{4}\right)}^{k}$. Here we have assumed that the hash function is one-to-one.

Note that in each read, errors are introduced with an error rate of *∈ *in the sequencing process. Even when we incorporate these errors, the above analysis will remain the same since if we have two random bases *a *and *b *and each is either kept the same or changed to another random base with probability *∈*, the probability that these two bases are equal will remain the same as 1/4.

**Example: **If *r *= 50 and *k *= 15, then the above probability is ≤ 2.33 × 10−6. For the same value of *r*, if *k *= 20, then this probability is ≤ 2.27 × 10−9.

In summary, if we choose *k *to be large enough then, for any read, the size of *C*(*R*) will be very nearly the same as that of T (*R*).

We also have to ensure that for any read *R*, each neighbor *R*' of *R *will fall into at least one bucket that *R *falls into. In other words, we have to show that *R *and *R*′ will share at least one *k*-mer. Let the length of the overlapping region between *R *and *R*' be *w*. There are *w *- *k *+ 1 *k*-mers in this region for each of *R *and *R*'. Let *x *and *y *be any such *k*-mers of *R *and *R*', respectively. Probability that *x *and *y *are the same is $p={\left[{\left(1-\in \right)}^{2}+\frac{1}{3}\in^{2}\right]}^{k}$. *Prob*[*x *≠ *y*] = 1 - *p*. Probability that *x *is not the same as any *k*-mer in the overlapping region in *R*' is (1 - *p*)^*w*-*k*+1^. Clearly, this statement assumes independence among the *k*-mers in the same read which may not hold. Such analyses have proven to yield some good guidelines in practice (see e.g., [18]). The probability that no *k*-mer in the overlapping region of *R *is the same as any *k*-mer in the overlapping region of *R*' is ${\left(1-p\right)}^{{\left(w-k+1\right)}^{2}}$. As a result, the probability that *R *and *R*' share at least one *k*-mer in the overlapping region is $1-{\left(1-p\right)}^{{\left(w-k+1\right)}^{2}}$. This probability can be made very large by choosing an appropriate value for *k*. If we do so, then for every read *R*, we will be able to identify a large fraction of its neighbors.

**Example: **Consider the case where *r *= 60, *w *= 40, *∈ *= 0.05, and *k *= 20. The value of *p *= 0.1309. Probability that *R *and *R*' share at least one *k*-mer in the overlapping region is ≥ 1 - 1.34 × 10−27. If *r *= 60, *w *= 40.*∈ *= 0.05, and *k *= 25, then this probability is ≥ 1 - 1.76 × 10−10.

Once we align the potential neighbors of *R *with *R *and prune those that are not likely to be neighbors and keep only valid neighbors, we perform error correction. If the number of neighbors we have identified for any read is large enough then the error correction will be effective. Let *q *be the number of neighbors available for a specific position of the read *R*. Then the number *e *of errors occurring in this position across all the neighbors is Binomially distributed with parameters *q *and *∈*. We want the number of errors to be strictly less than $\frac{q}{2}$. Using Chernoff bounds, $Prob\left[e>\left(1+\alpha \right)q\in \right]\leq \text{exp}\left(-\alpha^{2}q\in /3\right)$, for any fixed *α *> 0. For a choice of $\alpha =\left(\frac{0.4}{\in }-1\right)$, we get:

$$Prob\left[e>0.4q\right]\leq \text{exp}\left[-{\left(\frac{0.4}{\in }-1\right)}^{2}\frac{q\in }{3}\right].$$

**Example: **Consider the example of *q *= 20 and *∈ *= 0.05. The expected number of errors is 1. Probability that the number of errors is 10 or more is $\sum_{i=10}^{20}\left(\begin{array}{c} 20\\ i \end{array}\right)\in^{i}{\left(1-\in \right)}^{20-i}=1.134\times 10^{-8}$.

## Results

The effectiveness of our algorithm EC has been evaluated by comparing it with some of the state-of-the-art algorithms in this domain, namely, Racer, Musket, Coral, and Reptile. We have evaluated EC on a number of Illumina/Solexa datasets and compared the results with the aforementioned error correction algorithms. The simulation results show that our proposed algorithm is indeed very effective and competitive. More details follow.

### Datasets

We have employed both real and synthetic datasets in our evaluation. Real datasets used are Illumina-generated short reads of various lengths (see Table 1). The six experimental datasets listed in Table 1 have been taken from Sequence and Read Archive (SRA) at NCBI. Reference genomes are Sanger assembled bacterial genomes of various kinds. Although our error correction procedure is entirely *de novo*, the reference genome is necessary for evaluating the effectiveness of any error correction method. Synthetic datasets have been generated as follows. We have used various reference genomes for this purpose. We have generated reads from each reference genome. Specifically, each read was generated starting from a random position in the genome (see Table 2). To introduce errors in these synthetic datasets, we have changed each base in any read to some other base with error probabilities of 2%. A read length of 60 and a coverage of 50 have been used in D7-D12. Please note that *G_R_*, |*G*|, |*R*|, and |*r*| refer to accession number of the reference genome, genome length, total number of reads, and read length, respectively.

**Table 1:** Real Sequencing Datasets.

Dataset	Name	Accession	*G_R_*	|*G*|	|*r*|	|*R*|	Coverage
D1	*L. lactis*	SRR088759	NC 013656.1	2,598,144	36	4,370,050	60

D2	*E. coli*	SRR022918	NC 000913.1	4,771,872	47	6,740,651	68

D3	*E. coli*	SRR396536	NC 000913.2	4,639,675	75	3,453,957	55

D4	*B. subtilis*	DRR000852	NC 000964.3	4,215,606	75	1,744,210	31

D5	*E. coli*	SRR396532	NC 000913.2	4,639,675	75	4,341,061	70

D6	*L. interrogans L*	SRR353563	NC 004342.2	4,338,762	100	3,530,694	81

**Table 2:** Random Sequencing Datasets.

Dataset	Name	Accession	*G_R_*	|*G*|	|*R*|
D7	*L. lactis*	SRR088759	NC 013656.1	2,598,144	2,165,120

D8	*T. pallidum*	SRR361468	CP002376.1	1,139,417	9,495,14

D9	*E. coli*	SRR396536	NC 000913.2	4,639,675	3,868,043

D10	*B. subtilis*	DRR000852	NC 000964.3	4,215,606	3,513,005

D11	*P. aeruginosa*	SRR396641	NC 002516.2	6,264,404	5,220,336

D12	*L. interrogans L*	SRR353563	NC 004342.2	4,338,762	3,615,635

### Experimental setup

All the experiments were done on an Intel Westmere compute node with 12 Intel Xeon X5650 Westmere cores and 48 GB of RAM. The operating system running was Red Hat Enterprise Linux Server release 5.7 (Tikanga). To compile the C++ source code we used the g++ compiler (gcc version 4.6.1) with the -O3 option. Time was measured by taking the CPU clock time which gives the instruction level elapsed time a program takes.

### Evaluation metrics

To determine the effectiveness of any short read error correction algorithm, the corrected reads are mapped to the genome and the number of mismatches is counted. This is a procedure that is routinely used (see e.g., [1]). Although this procedure has some drawbacks (e.g., we have to align the reads with the reference genome with a certain number of mismatches), this is the best possible way to infer the accuracy of the error correction methods. In this context, we have used RMAP (v2.05) by [19]. It aligns short reads with a known genomic sequence by minimizing mismatches. For testing the accuracy we need to align as many corrected reads as possible so that the result will be correct with a high confidence. Keeping this in mind, we have allowed up to 10 mismatches per read for all of the datasets listed in Table 1. In brief: at first the error correction methods of interest are given the whole set of reads. After correction we align the reads over the genome of interest using RMAP within 10 mismatches and compute the performance metrics based on this alignment. Note that if we employ synthetic datasets, there is no need for mapping since we have information about true reads and erroneous reads.

A number of measures have been introduced in the literature for judging the performance of any error correction altorithm. True positives (TP) is a measure of how many erroneous bases have been corrected while false positives (FP) is the number of true bases that have been changed incorrectly. True negatives (TN) shows how many true bases remain unchanged while false negatives (FN) is the number of erroneous bases that have not been detected by the algorithm. Using these statistics we can define the following evaluation metrics:

1 *Sensitivity: *Sensitivity (also called the true positive rate, or the recall rate) measures the proportion of actual positives which are correctly identified as such (e.g., the percentage of sick people who are correctly identified as having the condition). In this context sensitivity is defined as: $Sensitivity=\frac{TP}{TP+FN}$.

2 *Specificity: *Specificity (sometimes called the true negative rate) measures the proportion of negatives which are correctly identified as such (e.g., the percentage of healthy people who are correctly identified as not having the condition). So, the specificity is: $Specificity=\frac{TN}{TN+FP}$.

3 *Accuracy: *Accuracy indicates the fraction of errors effectively removed from the experimental dataset. We can define it as follows: $Accuracy=\frac{TP-FP}{TP=FN}$. It is evident from the above definition that if the accuracy of an algorithm is large, then it is very effective in correcting errors. A negative value of accuracy indicates that the method of interest introduces more errors than it corrects.

4 *Erroneous base assignment (EBA)*: EBA is proposed in [6]. Let *b_e _*be the number of erroneous bases that are identified correctly by the error correction method but it replaces the erroneous bases with wrong bases. EBA is defined as follows: $EBA=\frac{b_{e}}{TP+b_{e}}$. Clearly, EBA reflects an algorithm's efficiency in correcting a base when it identifies this base to be erroneous. The lower this value the better is the algorithm.

5 *Cumulative Hamming Distance (CHD): *After aligning a read *R_i _*onto the genomic sequence, we calculate the Hamming distance *d_i _*between the aligned read and the corresponding sequence in genome. Adding all such *d_i _*for all the reads *R_i_*, we get *CDH*. It reflects how close the corrected reads are to a genomic sequence of the same species in terms of substitution errors.

6 *% Mapped Reads: *The fraction of reads from the entire space of reads aligned onto the reference genome with up to *d *mismatches.

### Parameters configuration

An algorithm always should tune its parameters with respect to a given dataset. Our algorithm has a set of parameters that are tuned automatically. Keeping this in mind we took the default parameter values for the different error correction methods that we have used for comparison:

• *Reptile: *Standard parameters.

• *Coral: *Standard parameters.

• *Racer: *Appropriate genome length of interest.

• *Musket: *Standard parameters.

• *EC: *No parameters to be selected. Parameters are empirically estimated based on an analysis of the input data. For example k varies from 14 to 17, Hamming distance ranges from 1 to 3, etc. The method has an interface where parameters can be fine tuned by the users if they are not satisfied with the results.

### Outcome

We have compared our algorithm with 4 other state-of-the-art algorithms based on both real and simulated reads. We have done extensive and rigorous experiments to realize that EC is indeed an effective and competitive error correction tool. Real sequencing data are taken from Sequence Read Archive (SRA) as described above. The results for the real sequencing datasets listed in Table 1 can be found in Table 3. The results for the synthetic datasets listed in Table 2 can be found in Table 4. We include erroneous base assignment (EBA) and cumulative hamming distance (CHD) measures as well. As mentioned previously, for simulated reads there is no need to consider the alignment of reads using any alignment.

**Table 3:** Performance evaluation on real sequencing datasets.

Dataset	Method	% Sensitivity	% Specificity	% Accuracy	% Mapped Reads	CPU Time (m)
D1	EC	88.59	99.99	85.78	95.75	5.76
	Racer	**96.98**	99.99	**96.87**	95.81	12.58
	Musket	80.99	99.99	80.94	**95.82**	**4.75**
	Coral	71.06	99.99	70.64	95.79	40.98
	Reptile	11.77	99.99	11.33	95.81	9.83

D2	EC	**94.44**	99.94	93.22	80.02	**12.10**
	Racer	93.92	99.99	**93.89**	**83.94**	13.93
	Musket	47.82	99.99	47.79	66.33	38.62
	Coral	33.68	99.99	33.22	65.32	141.64
	Reptile	44.76	99.99	44.73	67.51	34.02

D3	EC	**95.68**	99.97	**94.03**	**96.79**	**12.62**
	Racer	88.87	99.99	88.75	96.09	13.68
	Musket	69.50	99.99	69.43	94.09	19.00
	Coral	67.53	99.99	67.25	93.78	207.13
	Reptile	-	-	-	-	-

D4	EC	**94.89**	99.98	**93.41**	94.79	**4.65**
	Racer	93.42	99.99	93.30	**94.90**	6.40
	Musket	74.52	99.99	74.43	93.20	8.23
	Coral	74.40	99.99	74.07	92.54	28.47
	Reptile	-	-	-	-	-

D5	EC	**96.41**	99.97	**95.07**	**95.76**	18.27
	Racer	89.97	99.99	89.77	94.99	**14.38**
	Musket	63.64	99.93	63.58	91.44	27.22
	Coral	61.56	99.99	61.23	91.08	98.89
	Reptile	-	-	-	-	-

D6	EC	93.04	99.99	86.32	89.51	24.33
	Racer	**94.20**	99.99	**93.81**	**90.79**	12.63
	Musket	84.58	99.99	84.39	89.79	**11.32**
	Coral	89.34	99.99	83.28	90.14	233.33
	Reptile	-	-	-	-	-

Best results are shown in bold.

**Table 4:** Performatnce evaluation on simulated sequencing datasets having length 60 and coverage 50.

Dataset	Method	% Sensitivity	% Specificity	% Accuracy	EBA	CHD	CPU Time (m)
D7	EC	98.13	99.99	97.99	**2.51 × 10^−5^**	**52,281**	6.49
	Racer	**98.86**	99.91	94.43	9.55 × 10^−4^	1,47,171	**5.72**
	Musket	95.63	99.99	95.61	6.99 × 10^−5^	1,14,474	9.42
	Coral	93.47	99.99	92.76	1.08 × 10^−4^	1,88,584	23.02

D8	EC	98.11	99.99	97.79	6.88 × 10^−5^	25,238	2.91
	Racer	**99.44 **	99.98	98.34	2.11 × 10^−4^	**19,108**	**2.13**
	Musket	95.67	99.99	95.67	**2.02 × 10^−5^**	49,438	6.84
	Coral	93.46	99.99	93.15	5.16 × 10^−5^	78,162	9.57

D9	EC	98.05	99.98	97.10	1.50 × 10^−4^	**1,35,092**	12.26
	Racer	**98.92 **	99.95	96.36	5.57 × 10^−4^	1,71,542	**9.10**
	Musket	95.63	99.99	95.59	**7.87 × 10^−5^**	2,04,776	16.88
	Coral	93.45	99.98	92.53	1.42 × 10^−4^	3,47,258	38.01

D10	EC	98.14	99.99	97.78	5.99 × 10^−5^	**93,821**	11.12
	Racer	**99.00 **	99.96	97.13	4.28 × 10^−4^	1,22,798	**7.88**
	Musket	95.70	99.99	95.69	**3.12 × 10^−5^**	1,81,777	22.76
	Coral	93.51	99.99	93.07	6.32 × 10^−5^	2,92,345	34.21

D11	EC	98.08	99.99	97.69	**7.21 × 10^−5^**	**1,45,035**	19.64
	Racer	**98.12 **	99.58	77.23	4.23 × 10^−3^	14,46,476	**14.18**
	Musket	95.54	99.99	95.47	1.36 × 10^−4^	2,84,642	29.44
	Coral	93.48	99.98	92.75	1.10 × 10^−4^	4,54,814	59.33

D12	EC	**97.52 **	99.84	89.51	1.34 × 10^−3^	4,59,980	16.43
	Racer	97.27	99.69	82.19	2.96 × 10^−3^	7,82,370	**9.19**
	Musket	94.78	99.99	94.46	**5.76 × 10^−4^**	**2,42,375**	28.50
	Coral	93.31	99.91	88.79	7.00 × 10^−4^	4,88,658	50.91

Best results are shown in bold.

## Discussion

**Figure 1:**
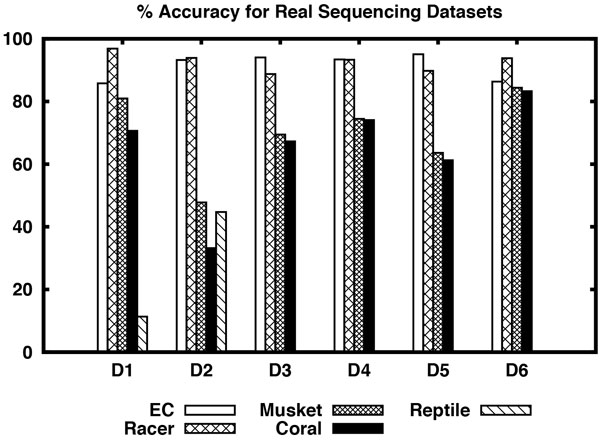
**% Accuracy of different algorithms including EC for real sequencing datasets D1-D6**.

**Figure 2:**
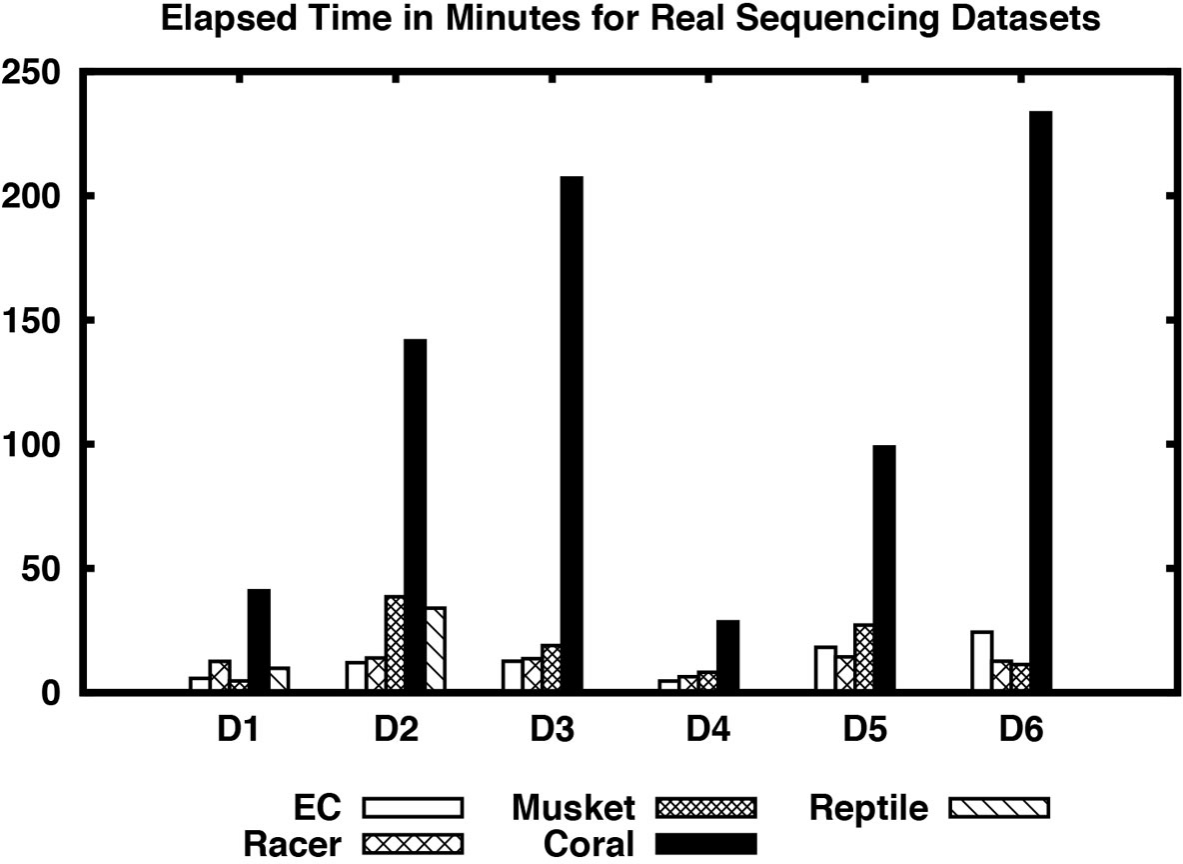
**Elapsed time of different algorithms including EC for real sequencing datasets D1-D6**.

Consider the results shown in Table 3 for real sequencing datasets. In D1 dataset, Racer performs better than all other algorithms including EC in terms of sensitivity and accuracy. Although the fraction of the mapped reads produced by Masket is better and also it takes less time to correct reads, its sensitivity and accuracy are very poor compared to Racer and EC. EC is comparable with Racer in D2 dataset and performs better than the rest of the algorithms. Although EC's accuracy and fraction of the mapped reads are slightly less than Racer, it clearly beats all the algorithms in terms of sensitivity and computation time. For D3-D5 datasets EC clearly performs better than all the algorithms including Racer with respect to sensitivity and accuracy. Its fraction of the mapped reads is slightly lower than Racer in D4 dataset. In D5 dataset EC's computation time is slightly greater than Racer. Overall EC is clearly the winner on D3-D5 datasets. On D6 dataset Racer beats every algorithm including EC. Please note that Reptile was not able to output corrected reads on D3-D6 datasets. Please see Figure 1 and 2 for a visual comparison of the algorithms on real sequencing datasets.

**Figure 3:**
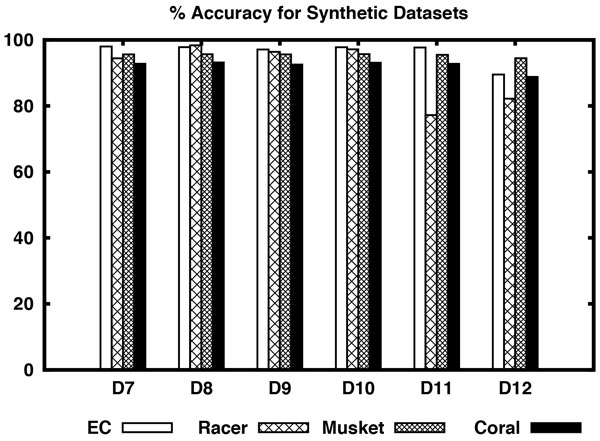
**% Accuracy of different algorithms including EC for synthetic datasets D7-D12**.

**Figure 4:**
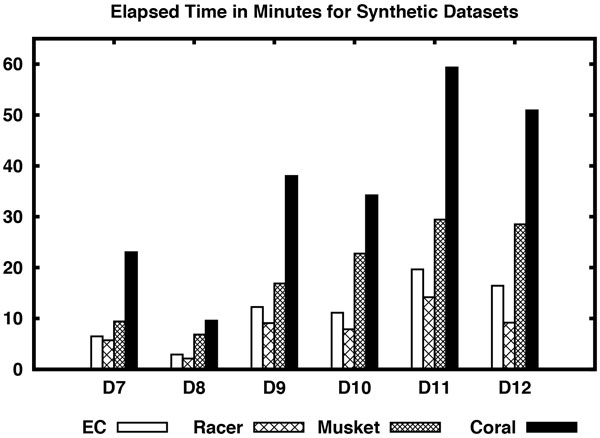
**Elapsed time of different algorithms including EC for synthetic datasets D7-D12**.

Now consider the results shown in Table 4 for synthetic datasets. On datasets D7 through D12, EC performs better than all the algorithms in terms of accuracy and CHD except for datasets D8 and D12. Its execution times are also comparable with Racer except for dataset D12. Although Musket performs better than all other algorithms in terms of EBA in most of the datasets, it performs poorly in terms of sensitivity, accuracy, etc. except for dataset D12. On D12 dataset, Musket performs better than the rest with respect to accuracy, EBA, CHD, and execution time. Reptile could not be run on the simulated datasets as it needs quality information of the bases. Please see Figure 3 and 4 for a visual comparison of the algorithms on simulated datasets.

## Conclusions

In this article we have proposed an efficient, scalable, and robust error correction algorithm for correcting short reads. The steps of EC can be broken into three independent tasks. At first it builds *k*-mers and hashes the *k*-mers into hash tables. Using these hash tables it finds the neighbors of each of the reads. Each read is then corrected using the neighbors of the read. We have introduced a number of techniques to correct reads more effectively. We have compared our algorithm with four state-of-the-art algorithms based on both real and simulated reads. Our experiments reveal that EC is indeed effective and competitive. At this time EC can only handle substitution errors. In future we plan to develop similar techniques to handle insertion and deletion errors also.

## Competing interests

The authors declare that they have no competing interests.

## Funding

This work has been supported in part by the following grants: NIH R01-LM010101 and NSF 1447711.

## Authors' contributions

SR and SS have developed the algorithms. SS has implemented the algorithms. Results have been analyzed by SS and SR. The paper has been written by SR and SS.

## Declarations

Publication of this article was funded by the University of Connecticut.

This article has been published as part of *BMC Bioinformatics *Volume 16 Supplement 17, 2015: Selected articles from the Fourth IEEE International Conference on Computational Advances in Bio and medical Sciences (ICCABS 2014): Bioinformatics. The full contents of the supplement are available online at http://www.biomedcentral.com/bmcbioinformatics/supplements/16/S17.
